# Orthopedic Team Surgeons in Major Professional Sports: An Analysis of Affiliation With the Top 10 Sports Medicine Fellowship Programs and Implications for Leadership and Diversity

**DOI:** 10.7759/cureus.54259

**Published:** 2024-02-15

**Authors:** Robert Wood, Jorge Perera, Jacqueline Krumrey, Christopher McCrum

**Affiliations:** 1 Orthopedics, Good Samaritan Regional Medical Center, Corvallis, USA; 2 Orthopedics, Samaritan Health Services, Corvallis, USA; 3 Orthopedics and Traumatology, Good Samaritan Regional Medical Center, Corvallis, USA; 4 Orthopedic Sports Medicine, Samaritan Health Services, Corvallis, USA

**Keywords:** sports medicine fellowship, fellowship training, fellowship match, san francisco match, arthroscopy surgeon, program directors, fellowship programs, orthopedic sports medicine, sports medicine, orthopedics

## Abstract

This paper examines the correlation between orthopedic team surgeons in major professional sports and their affiliation with the top 10 sports medicine fellowship programs. With a growing trend in post-residency fellowship training, particularly in sports medicine, the study focuses on the implications of fellowship program choice for aspiring major professional sports team physicians. By analyzing data from Major League Baseball (MLB), the National Basketball Association (NBA), the National Football League (NFL), and the National Hockey League (NHL), the research reveals that 61 of 124 (49.19%) team surgeons graduated from the top 10 sports medicine fellowship programs. The results identify a noticeable pipeline effect in professional sports, where teams often hire graduates from a select number of esteemed fellowship programs. The study suggests that choosing a fellowship program from the top 10 list may enhance the prospects of becoming a major league team surgeon. Additionally, our results found a significant gender disparity among team surgeons, with only two (1.6%) of all major professional team physicians being women. This emphasizes the imperative for diversity improvement in orthopedic sports medicine. In conclusion, the research underscores the impact of top-tier fellowship programs on professional team surgeons, with implications for aspiring sports medicine physicians and a call for addressing gender disparities.

## Introduction

The trend of pursuing subspecialty fellowship training in orthopedic surgery post-residency has experienced a notable rise, with the percentage of fellowship applicants increasing from 76% to 90% between 2003 and 2013 [1]. Within the orthopedic subspecialties, sports medicine fellowships have emerged as highly competitive. Mulcahey et al.'s study covering 2010-2017 revealed that the number of sports medicine fellowship applicants consistently exceeded available positions [2]. This can be contrasted with a 2014 study by Daniels et al., which reported an overall surplus of positions across the nine orthopedic subspecialties [3]. This further underscores the popularity and competitive nature of sports medicine fellowship training.

As the pursuit of orthopedic fellowship training becomes more commonplace, attention is increasingly directed toward the characteristics of fellowship programs and their perceived prestige [4]. This is particularly relevant for those aspiring to become team physicians, as the choice of fellowship program may influence the likelihood of securing a prominent attending position [5]. The role of team physicians and their responsibilities have evolved significantly since the 19th century. Beyond athlete care, team physicians now handle administrative, legal, and communication duties, varying based on the athlete's level. Despite these increasing demands, team physicians must continue contributing to sports medicine through technical advances, teaching, and research. Their unique access to athletes at all levels of sport positions them as valuable contributors to advancements in the field. This includes innovations in anterior cruciate ligament reconstruction, shoulder arthroscopy, and ulnar collateral ligament reconstruction surgery [6]. 

Recognizing the pivotal role of team physicians, particularly orthopedic team physicians, in advancing sports medicine, understanding their contributions to teaching and research at all levels of sport becomes paramount. This understanding is crucial for appreciating the collective impact of team physicians on the evolution and progress of sports medicine. Among the most meaningful contributors to the advancement of sports medicine are surgeons who serve as team surgeons for professional teams and those who work and train at well-respected academic institutions. With these requirements, obtaining a major league team physician job position has become highly desirable. Many factors play a role in obtaining this position, such as educational background and fellowship training. A recent survey established the perceived top 10 most esteemed orthopedic sports medicine fellowship programs [7]. Similarly, previous studies have identified potential pipelines for graduates from the top 10 sports medicine fellowship programs to enter faculty roles at prestigious institutions [8]. The purpose of this study was to look for comparable patterns in team surgeons for American major professional sports. The authors hypothesized that most physicians serving as head team surgeons for professional sports teams would be graduates of one of the top 10 sports medicine fellowship programs.

## Materials and methods

**Figure 1:**
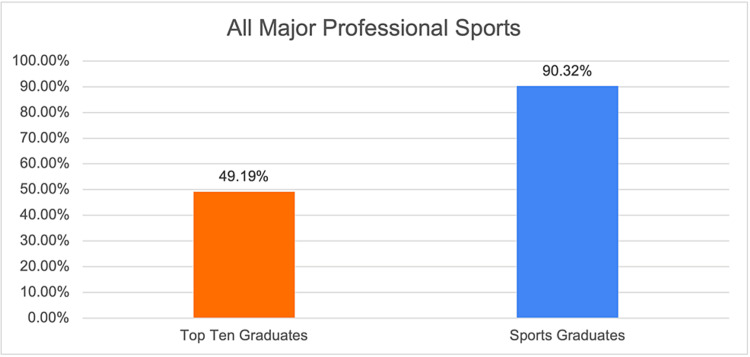
Graphical representation of the proportion of team surgeons in professional sports who have trained at the top 10 sports medicine fellowship programs and orthopedic sports medicine programs in general.

**Figure 2:**
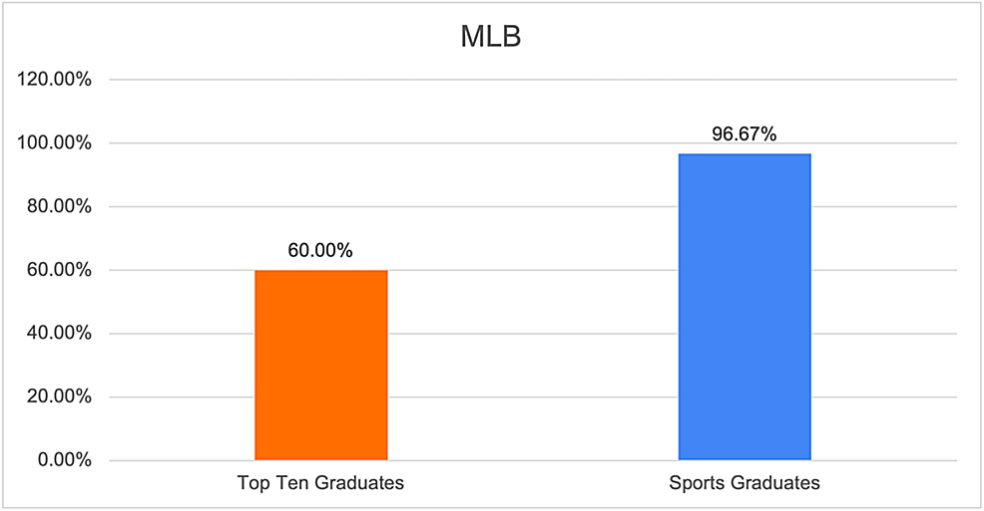
Graphical representation of the proportion of team surgeons in the MLB who have trained at the top 10 sports medicine fellowship programs. MLB: Major League Baseball.

**Figure 3:**
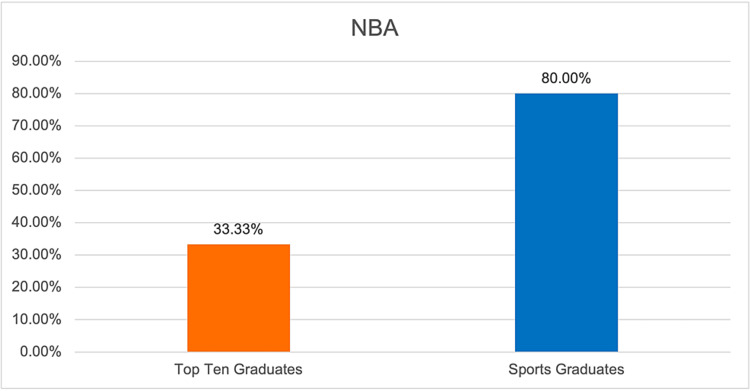
Graphical representation of the proportion of team surgeons in the NBA who have trained at the top 10 sports medicine fellowship programs. NBA: National Basketball Association.

**Figure 4:**
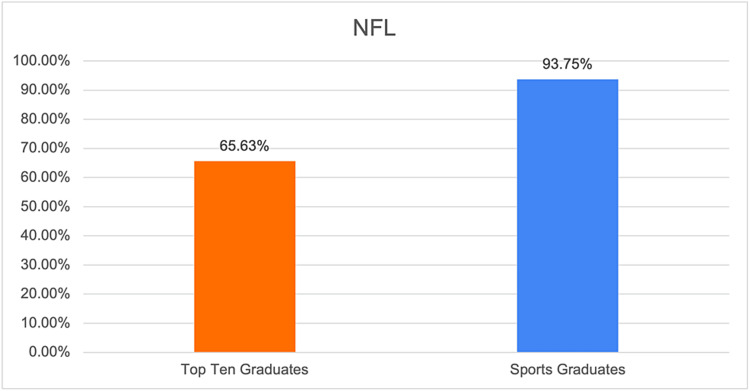
Graphical representation of the proportion of team surgeons in the NFL who have trained at the top 10 sports medicine fellowship programs. NFL: National Football League.

**Figure 5:**
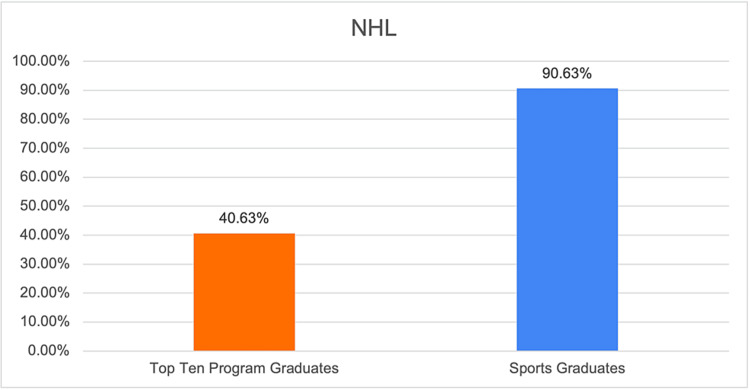
Graphical representation of the proportion of team surgeons in the NHL who have trained at the top 10 sports medicine fellowship programs. NHL: National Hockey League.

This study used the top 10 sports medicine fellowship programs previously identified by other published studies, as depicted in Table 1 [7,8]. The training programs of head team surgeons for teams in the four most popular professional sports by viewership in the United States, Major League Baseball (MLB) the National Basketball Association (NBA), the National Football League (NFL), and the National Hockey League (NHL), were identified by searching team websites in November 2023. The number of team surgeons who completed an accredited orthopedic sports medicine fellowship was then determined. The gender of each team surgeon was also identified. Additionally, the proportion of team surgeons who completed fellowship at the top 10 sports medicine fellowship programs was calculated. Descriptive statistics were then used to report the proportion of team surgeons who completed fellowship at the top 10 sports medicine fellowship programs for all four professional sports leagues and for each league individually (Figures 1-5).

**Table 1:** The top 10 orthopedic sports medicine fellowship programs, as ranked by fellowship applicants. Source: Tanguilig et al. [7,8].

Rank	Program
1	Steadman Philippon Research Institute Program
2	Rush University Medical Center Program
3	Hospital for Special Surgery/Cornell Medical Center Program
4	OrthoCarolina Sports Medicine, Shoulder, & Elbow Program
5	Cedars-Sinai Kerlan-Jobe Orthopaedic Clinic Program
6	American Sports Medicine Institute (St. Vincent’s) Program
7	University of Pittsburgh/UPMC Medical Education Program
8	Steadman Hawkins Clinic of the Carolinas Program
9	Steadman Hawkins Clinic Denver Program
10	Duke University Hospital Program

Data were obtained from team websites and directories from publicly available league resources. The team surgeon was successfully identified for all 30 MLB teams and each of the 32 NFL teams. There was one team for which a team surgeon could not be identified in both the NBA and NHL. After properly identifying the team surgeons, professional websites were searched to determine their specific subspecialty and the program they graduated from. Descriptive statistics were then used to calculate the proportions of surgeons who attended the top 10 sports medicine fellowship programs for each major professional sport as well as all professional sports teams overall (Table 2).

**Table 2:** Percentage of team surgeons who completed fellowships at the top 10 sports medicine fellowship programs by league and overall, in professional sports. MLB: Major League Baseball; NBA: National Basketball Association; NFL: National Football League; NHL: National Hockey League.

League	Percentage of graduates from the top 10 sports medicine fellowship programs
MLB	60%
NBA	33.33%
NFL	65.63%
NHL	40.63%
Total	49.19%

## Results

Overall, 118 of 124 (95.2%) team surgeons listed their subspecialty as sports medicine. The remaining subspecialties are listed in Table 3. In all professional sports, 61 of 124 (49.19%) team surgeons were graduates from the top 10 sports medicine fellowship programs. The most frequently attended the top 10 sports medicine fellowship programs was the Hospital for Special Surgery, with 17 graduates serving as team surgeons for professional teams. This was followed closely by the Kerlan-Jobe Institute, with 13 graduates, and the University of Pittsburgh Medical Center, with 11 graduates. Notably, there were no Duke University graduates identified as current team surgeons. All other top 10 programs had at least one graduate serving in a major professional team surgeon role. When examining the gender of the current team surgeons, only two of 124 team surgeons identified as female representing 1.6% of the current major professional team surgeons. 

**Table 3:** Number of team surgeons who list a subspecialty other than sports by subspecialty type.

Specialty	Number of Surgeons
Trauma	2
Shoulder and elbow	1
Upper extremity	3

## Discussion

Based on the results of our study, we found that the majority of orthopedic surgeons listed as team surgeons for major sports teams completed their fellowship at the top 10 sports medicine fellowship programs. There was some variability based on the specific professional sport, with the highest rates of top 10 graduates serving as team surgeons in the NFL at 65.63% or 21 out of 32 listed team surgeons (Table 2). Conversely, the NBA had the lowest percentage of team surgeons from the top 10 sports medicine fellowship programs at 33.33% or 10 out of 30 surgeons. 

This study showed that many teams tend to employ graduates from a small number of highly esteemed sports fellowship programs. Similar studies have found that graduates of top 10 programs often serve in other prestigious roles, such as fellowship program directors [9-11]. Also, this study reveals a comparable pipeline phenomenon in professional sports, as teams employ graduates of a small number of highly esteemed sports fellowship programs. Similar patterns have been described in residency and fellowship programs [8]. Prior publications have hypothesized that this pipeline effect may be due to comfortability and bias towards the reputations of attendings who have completed training at a top institution [8]. Therefore, sports medicine surgeons who have a desire to serve as team surgeons for professional teams may wish to match at one of the fellowship programs included in the top 10 list.

In examining diversity among the surgeons serving as team surgeons, there was a significant gender disparity. There were only two female surgeons listed as team surgeons in all of the major professional sports, representing 1.6% of team surgeons. Prior studies have found similar disparities in the numbers of female orthopedic surgery residents, practicing orthopedic surgeons, and fellowship program directors [11,12]. This exemplifies the continued need for improvement in diversity in orthopedic surgery and specifically for orthopedic sports medicine. A concerted effort should be made to study the underlying reasons that have led to the gap in female representation among orthopedic sports medicine physicians.

This study did have several limitations that should be considered. First, the list of the top 10 sports medicine fellowship programs is based on a single survey study and is entirely subjective. Further, the reputation of a fellowship program is fluid and can change based on a variety of factors that could not be accounted for in our study. Additionally, the list of team surgeons was obtained through publicly available resources, which may not always be kept up-to-date. Further, the data analyzed were only from the United States and may not reflect patterns in other countries. Also, the reasons for the variability of the proportions of top 10 graduates among different professional sports leagues were unable to be determined. Finally, the reason for teams hiring specific surgeons cannot be ascertained from this study, and thus, no correlative or causative link can be established between the fellowship program and the ability to attain professional team surgeon status. 

## Conclusions

Nearly half of the surgeons serving in the team surgeon role at the professional level have received fellowship training at the top 10 orthopedic sports medicine fellowship programs. This phenomenon represents a trend toward pipeline training systems similar to those that exist for orthopedic surgery program directors and surgeons involved in high-level academia. Additionally, there are significantly fewer female surgeons serving as team surgeons for professional teams when compared to their male counterparts. The results of this paper underscore the need for stronger emphasis on increasing diversity in all realms, including gender, ethnicity, and training backgrounds.
